# Characterization of the photoelectron circular dichroism of fixed-in-space molecules through an asymmetry of the ionic potential

**DOI:** 10.1063/4.0000300

**Published:** 2025-04-18

**Authors:** Eric Kutscher, Anton N. Artemyev, Philipp V. Demekhin

**Affiliations:** Institut für Physik und CINSaT, Universität Kassel, Heinrich-Plett-Str. 40, 34132 Kassel, Germany

## Abstract

Photoelectron circular dichroism (PECD) in the ionization of chiral molecules by circularly polarized radiation is a well-established tool for chiral recognition in the gas phase. The effect consists in a forward-backward asymmetry in angular emission distributions of photoelectrons with respect to the light propagation direction, which survives averaging over molecular orientations. Its magnitude is governed by the ability of the outgoing photoelectron to probe an asymmetry of the ionic potential by multiple scattering effects, and it can be significantly enhanced by fixing molecular orientation in space. Even achiral fixed-in-space molecules can exhibit such a forward-backward asymmetry in the photoemission. In the present work, we establish a qualitative correspondence between the PECD in one-photon ionization of fixed-in-space molecules and a degree of the asymmetry of their ionic potential. For this purpose, we introduce an enantiosensitive dichroic characteristic of the ionic potential, which describes a physical mechanism behind the forward-backward asymmetry in the photoemission from fixed-in-space molecules ionized by circularly polarized light. This characteristic, as a function of molecular orientation angles, can be compared to the respective PECD landscape. The present findings are exemplified by several applications to achiral and chiral species.

## I. INTRODUCTION

The fact that interaction of chiral molecules with circularly polarized light depends on its helicity is naturally utilized in almost all chiral recognition techniques. Because of its weak magnitude, circular dichroism (CD) in the absorption is usually utilized in the condensed or liquid phases.^1^ Since its first experimental verification,^2,3^ another, considerably stronger chiroptical effect, which is known as photoelectron circular dichroism (PECD), is nowadays routinely utilized in the gas phase.^4–7^ PECD was predicted theoretically almost 50 years ago^8–10^ for one-photon ionization of randomly oriented chiral molecules, and it consists in the forward-backward asymmetry in photoelectron emission distributions with respect to the light propagation directions. The effect is also present in the multiphoton ionization,^11,12^ and it is universal with respect to the photoionization regime.^13,14^

For randomly oriented molecules, the strengths of the effect is reduced to a few percent of the total ionization signal, since any averaging over molecular orientations causes loss and, thus, reduction of the chiral response.^8–10^ Therefore, fixing a molecular orientation in space by any means results in considerably stronger forward-backward asymmetries.^15–21^ Indeed, fixing a molecule only partly in space (uniaxial orientation) increases this asymmetry to about 10%–30%,^16,17,19^ while the PECD of a fully fixed-in-space molecule can be increased to 50%–80%.^15,16,18,21^ A dynamical alignment during the multiphoton ionization of chiral molecules influences the magnitude of PECD as well.^22,23^ Such a pronounced contrast improves the sensitivity for chiral recognition. Even achiral molecules being fixed in space exhibit strong forward-backward asymmetries (the so-called *apparent* PECD^24^) which, of course, vanish upon orientation averaging. Thus, one can speak of different contributions to the PECD of fixed-in-space molecules: the intrinsic asymmetry provided by the molecule itself and the orientation-induced extrinsic asymmetry introduced to the system by an experimental geometry.^24^

A relevant question immediately arises: At which orientations of a chiral molecule can one expect larger PECD and why? A recent elegant theoretical approach^25^ has established a generalized perspective on chiral measures, formulated propensity rules for the PECD,^26,27^ and performed a characterization of ultrafast molecular chirality.^28,29^ In addition, this approach allows one to draw several important and general conclusions on different enantiosensitive observables on the base of a geometric propensity field.^30^ However, detailed characterization of this field relies in the approach on quantum mechanical calculations of the respective electric-dipole photoionization transition amplitudes. Can one predict distinct molecular orientations for largest PECD without performing calculations of the PECD itself or any quantum mechanical consideration of the photoionization process? For instance, several works suggest quantification of a degree of intrinsic chirality based only on geometrical properties,^31–35^ or on properties of a molecular potential and density matrix.^36^ The latter approach proposes a route to systematically tune an artificial chiral potential to maximize the respective PECD for randomly oriented molecules.

Since a magnitude of the PECD, including its apparent part, is governed by the ability of the outgoing photoelectron to probe the asymmetry of the molecular potential by multiple scattering effects, it is worthwhile to search for a qualitative correspondence of the PECD of fixed-in-space molecules and asymmetry properties of the electronic potential of a molecular ion. This seems to be a viable route, since the ionic potential “knows everything” about asymmetries of initial bound and final continuum electronic states of the photoionization process. For this purpose, we perform in the present work an analysis of the ionic potential and establish its simplest possible characteristic, which can be compared to the one-photon PECD computed at different molecular orientations. This characteristic allows one to suggest molecular orientations with maximal PECD without considering photoionization of a molecule by *ab initio* theory.

The paper is organized as follows. In Sec. II, we discuss characterization of the PECD of fixed-in-space molecules and introduce respective characteristic of the ionic potential. In Sec. III, we report several applications of the present findings to achiral and chiral molecules. We conclude in Sec. IV with a brief summary and outlook.

## II. THEORETICAL APPROACH

In this section, we introduce the electronic potential of the molecular ion (Sec. II A), define the PECD of fixed-in-space molecules (Sec. II B), and perform characterization of the potential (Sec. II C). Atomic units are used throughout this manuscript.

### A. Electronic potential of the molecular ion

We start with the local potential, generated by the molecular ion for a single-active photoelectron. It includes contributions from all nuclei and remaining electrons and defines the initial bound and final continuum states of the photoelectron via a solution of the time-independent Schrödinger equation. In the molecular frame of reference, it has the following single-center representation in spherical coordinates:

$$V^{0,0,0}({\vec{r}}^{\;\prime })=\sum_{LM}V_{LM}^{0,0,0}(r^{\prime })\;Y_{LM}(\theta^{\prime },\varphi^{\prime }).$$ (1)

Here, the superscripts {α=0,β=0,γ=0} denote the three orientation Euler angles, at which the molecular and laboratory frames of references coincide. The Euler angle γ describes rotation around the laboratory z axis, α around the molecular $z^{\prime }$ axis, and β is the angle between the two axes. This potential $V^{0,0,0}({\vec{r}}^{\;\prime })$ can be generated at any fixed internuclear geometry by standard quantum chemistry codes. Assuming moderate nuclear dynamics during photoionization, an equilibrium geometry of a molecule in its neutral electronic ground state is a natural choice. In addition, similarly to our previous works,^37–41^ we always set the center of the molecule to the center of the total charge distribution of the ion. On the one hand, this improves convergences of the expansion (1) over indices *LM* and, thus, of the numerical solution of the Schrödinger equation over the angular momentum quantum numbers ℓm of the photoelectron wave packet. On the other, this eliminates the permanent dipole moment of the ion and excludes its impact on the asymmetry of the potential. By this unique choice, the center of a molecule is set inside a localization region of the initial bound electronic state, which was ionized (i.e., a region where the photoelectron was *born*).

With the help of the Wigner rotation matrices $D_{ij}^{\ell }$, the known expansion functions $V_{LM}^{0,0,0}(r^{\prime })$ allow one to generate this potential at an arbitrary molecular orientation {α,β,γ} by applying the following transformation:

$$V_{LM}^{\alpha ,\beta ,\gamma }(r)=\sum_{M^{\prime }}D_{M^{\prime }M}^{L}(\alpha ,\beta ,\gamma )\;V_{LM^{\prime }}^{0,0,0}(r^{\prime }).$$ (2)

Using properties of the rotation matrices,^42^ it is straightforward to demonstrate that, for each enantiomer, the expansion functions (2) obey the following asymmetry rule:

$$V_{LM}^{\alpha ,\beta ,\gamma }(r)={\left(-1\right)}^{L+M}{\left\{V_{LM}^{\alpha +\pi ,\pi -\beta ,-\gamma }(r)\right\}}^{*}.$$ (3)

As the next step, we define an interrelation between the two enantiomers (R and S), which, in the present work, are taken as the mirror images of each other in the $x^{\prime }z^{\prime }$-plane (i.e., we mirror $y^{\prime }\to -y^{\prime }$ in the potential),

$${\left[V^{0,0,0}(r^{\prime },\theta^{\prime },\varphi^{\prime })\right]}_{R}={\left[V^{0,0,0}(r^{\prime },\theta^{\prime },-\varphi^{\prime })\right]}_{S}.$$ (4)

From this definition, it follows that the expansion functions (2) for two enantiomers are interrelated as follows:

$${\left[V_{LM}^{\alpha ,\beta ,\gamma }(r)\right]}_{R}={\left[{\left\{V_{LM}^{-\alpha ,\beta ,-\gamma }(r)\right\}}^{*}\right]}_{S}.$$ (5)

Finally, for achiral molecules, we set the molecular frame such that a molecule coincides with itself upon the $y^{\prime }\to -y^{\prime }$ mirroring, which is always possible, except of a special case of achiral molecules which have no mirror plane (e.g., meso-tartaric acid with two apparent chiral centers). With this choice, ionic potentials (1) of the two mirror images of an achiral molecule are identical (R=S).

### B. PECD of fixed-in-space molecules

In a general case, the three-dimensional laboratory-frame photoelectron angular emission distribution from a molecule, which is fixed in space at orientation Euler angles {α,β,γ} and ionized by circularly polarized light with a positive (+) or negative (−) helicity, is given by the following expansion over the spherical functions:^43^

$$W^{\pm }(\vec{k},\alpha ,\beta ,\gamma )=\sum_{LM}W_{LM}^{\pm }(k,\alpha ,\beta ,\gamma )\;Y_{LM}(\theta_{k},\varphi_{k}).$$ (6)

Here, $\vec{k}$ is the photoelectron momentum given by its kinetic energy $\varepsilon =k^{2}/2$ and two laboratory-frame emission spherical angles $\left\{\theta_{k},\varphi_{k}\right\}$. The polar emission angle $\theta_{k}$ is defined with respect to the laboratory z axis, which coincides with the direction of the propagation of light. For circularly polarized light, which is axially symmetric along its propagation direction, a definition of the origin of the azimuthal angle $\varphi_{k}$ is irrelevant (see also below). Therefore, for simplicity, it was suggested in Ref. 44 to integrate the three-dimensional distribution (6) over the orientation Euler angle γ, which describes rotation of the molecular frame around the laboratory z axis. This integration can be performed analytically, and it recovers axial symmetry of the photoemission probability from a fixed-in-space molecule, by eliminating all expansion terms with M≠0. As a consequence, the resulting two-dimensional photoelectron angular distribution becomes independent of the azimuthal angle $\varphi_{k}$, and, for a given kinetic energy ε (momentum *k*), reduces to a simplified expansion over the Legendre polynomials,^15^

$$W^{\pm }(\theta_{k},\alpha ,\beta )=\sum_{L}b_{L}^{\pm }(\alpha ,\beta )P_{L}(\text{cos}\;\theta_{k}),$$ (7)

with $b_{L}^{\pm }=W_{L0}^{\pm }\sqrt{(2L+1)/4\pi }$.

In order to represent the PECD for each molecular orientation and light helicity by a single value, we use the strategy proposed in Refs. 45 and 46 and introduce the difference between the photoemission yields in the forward ( $F:\;0\leq \theta_{k}\leq \frac{\pi }{2}$) and backward ( $B:\;\frac{\pi }{2}\leq \theta_{k}\leq \pi$) hemispheres relatively to half of the total yield, averaged over all molecular orientations,

$$\begin{array}{c} FB^{\pm }(\alpha ,\beta )=\frac{1}{\left\langle b_{0}\right\rangle }[\int_{F}W^{\pm }(\theta_{k},\alpha ,\beta )\;\text{sin}\;\theta_{k}d\theta_{k}\\ -\int_{B}W^{\pm }(\theta_{k},\alpha ,\beta )\;\text{sin}\;\theta_{k}\;d\theta_{k}], \end{array}$$ (8)

with $\left\langle b_{0}\right\rangle$ defined as

$$\begin{array}{c} \langle b_{0}\rangle =\frac{1}{4\pi }\iint \left[\frac{1}{2}\int_{F+B}W^{\pm }(\theta_{k},\alpha ,\beta )\;\text{sin}\;\theta_{k}\;d\theta_{k}\right]\text{sin}\;\beta \;d\alpha \;d\beta =\frac{1}{4\pi }\iint b_{0}^{\pm }(\alpha ,\beta )\;\text{sin}\;\beta \;d\alpha \;d\beta , \end{array}$$ (9)

which, in the electric-dipole approximation, is independent of the light helicity. Using Eq. (7), we arrive at the following simplified expression for this forward-backward asymmetry:^15^

$$\begin{array}{c} FB^{\pm }(\alpha ,\beta )=\frac{1}{\left\langle b_{0}\right\rangle }\sum_{L_{\text{odd}}}b_{L}^{\pm }(\alpha ,\beta )D_{L}=\frac{1}{\left\langle b_{0}\right\rangle }\\ \left[b_{1}^{\pm }(\alpha ,\beta )-\frac{b_{3}^{\pm }(\alpha ,\beta )}{4}+\frac{b_{5}^{\pm }(\alpha ,\beta )}{8}-\frac{5b_{7}^{\pm }(\alpha ,\beta )}{64}\ldots \right], \end{array}$$ (10)

with the recursive coefficients $D_{L+2}=-\frac{L}{L+3}D_{L}$ and $D_{1}=1$.^15,45,46^

For the present discussion, we notice that the odd coefficients, entering the expansion (10), satisfy the following asymmetry property:^44^

$$b_{2L+1}^{+}(\alpha ,\beta )=-b_{2L+1}^{-}(\alpha +\pi ,\pi -\beta ).$$ (11)

With the help of expansion (10) for the forward-backward asymmetry, we now define the PECD of fixed-in-space molecules, represented by a single value as a function of the molecular orientation,^15^

$$\text{PECD}(\alpha ,\beta )=FB^{+}(\alpha ,\beta )-FB^{-}(\alpha ,\beta ).$$ (12)

Being integrated over all molecular orientations, this dichroic asymmetry PECD(α,β) yields the well-known value of the $\text{PECD}=2\langle b_{1}^{+}\rangle /\langle b_{0}\rangle =2\beta_{1}$ for randomly oriented molecules. According to Eq. (11), it has the following symmetry property for each enantiomer:

PECD(α,β)=PECD(α+π,π−β). (13)

Because of the present definition of the enantiomers via Eqs. (4) and (5), these PECD(α,β) obtained for two opposite enantiomers are interrelated as follows:

$$\text{PEC}D_{R}(\alpha ,\beta )=-\text{PEC}D_{S}(-\alpha ,\beta ).$$ (14)

The expansion coefficients $b_{L}^{\pm }(\alpha ,\beta )$ from Eq. (7), which are required to calculate the forward-backward characteristics (10), were computed in the present work for different molecules using the time-dependent single-center (TDSC) method and code.^15,37–41^ In order to generate the orientation-dependent characteristics, we used the following strategy. First, numerical calculations were carried out for the initial orientation angles {α=0,β=0,γ=0}, i.e., when the laboratory and molecular frames coincide. Details of such calculations can be found in our previous works.^15,37–41^ Using photoionization transition amplitudes, computed for all light polarizations at the initial orientation of a molecule (i.e., in the molecular frame), the respective coefficients $b_{L}^{\pm }(\alpha ,\beta )$ were then generated at each molecular orientation via an analytical expression derived in Appendix A.

### C. Characterization of the potential

We now characterize the electronic potential of a molecular ion, introduced in Sec. II A. A microscopic mechanism behind the PECD effect is usually exemplified by a mechanical analogy of a nut on a thread,^4,16,17^ where a molecular electronic structure acts as the gearbox and transforms the rotational motion of the electric field vector in a translational motion of the photoelectron flux. On average over molecular orientations, such an effect survives only for chiral molecules, since a mirror image of an achiral molecule, which provides an opposite effect, can be created by a rotation, and thus compensates the effect completely. It is, therefore, meaningful to search for a characteristic of the ionic potential of a fixed-in space molecule, χ(α,β), which is responsible for coupling of the rotational and translational motions of the photoelectron. Since, as can be anticipated, we would like to compare such a characteristic with the PECD(α,β) asymmetry, it should obey similar properties [Eqs. (13) and (14)], i.e., satisfy the following identities:

χ(α,β)=χ(α+π,π−β), (15)

$$\chi_{R}(\alpha ,\beta )=-\chi_{S}(-\alpha ,\beta ).$$ (16)

In addition, χ(α,β) needs to be dichroic, i.e., it has to incorporate an information of the sense of rotation associated with the forward and backward translations. A simplest single-valued characteristic of the potential of a fixed-in-space molecule can be constructed by subtracting the two relative integral differences between the terms of the potentials, which are sensitive to clockwise and counterclockwise rotations, one obtained for the forward and another for the backward hemispheres,

$$\begin{array}{c} \chi (\alpha ,\beta )=\text{Im}\{\frac{{\left[V^{\alpha ,\beta }(\vec{r},↻)-V^{\alpha ,\beta }(\vec{r},↺)\right]}_{F}}{\left|{\tilde{V}}_{00}\right|}\\ -\frac{{\left[V^{\alpha ,\beta }(\vec{r},↻)-V^{\alpha ,\beta }(\vec{r},↺)\right]}_{B}}{\left|{\tilde{V}}_{00}\right|}\}. \end{array}$$ (17)

Note that the function in the braces is purely imaginary by its construction. Alternatively, by reordering the terms in Eq. (17), one can arrive at another definition, which is common for the PECD:^45,46^ It is constructed by subtracting the two relative integral differences between the forward and backward hemispheres, one obtained for the terms of the potential that are sensitive to clockwise and another to counterclockwise rotations.

The respective rotation-sensitive parts of the potential can be interrelated with its expansion terms $V_{LM}^{\alpha ,\beta ,\gamma }(r)$ with the positive M>0 (for ↻) and negative M<0 (for ↺) index. Thereby, the difference of these rotation-sensitive parts of the potential, which is present in the nominators of Eq. (17), reads

$$\begin{array}{c} V^{\alpha ,\beta ,\gamma }(\vec{r},↻)-V^{\alpha ,\beta ,\gamma }(\vec{r},↺)=\sum_{L,M>0}V_{LM}^{\alpha ,\beta ,\gamma }(r)Y_{LM}(\theta ,\varphi )\\ -\sum_{L,M<0}V_{LM}^{\alpha ,\beta ,\gamma }(r)Y_{LM}(\theta ,\varphi ). \end{array}$$ (18)

This purely imaginary difference of the two complex-conjugate quantities can easily be simplified to

$$\begin{array}{c} V^{\alpha ,\beta ,\gamma }(\vec{r},↻)-V^{\alpha ,\beta ,\gamma }(\vec{r},↺)\\ =2i\sum_{L,M>0}\text{Im}\left[V_{LM}^{\alpha ,\beta ,\gamma }(r)Y_{LM}(\theta ,\varphi )\right]\\ =2i\sum_{L,M>0}\text{Re}\left\{V_{LM}^{\alpha ,\beta ,\gamma }(r)\right\}\text{Im}\left\{Y_{LM}(\theta ,\varphi )\right\}\\ +2i\sum_{L,M>0}\text{Im}\left\{V_{LM}^{\alpha ,\beta ,\gamma }(r)\right\}\text{Re}\left\{Y_{LM}(\theta ,\varphi )\right\}. \end{array}$$ (19)

According to the definition (17), this difference has now to be averaged over the forward ( ${\left[\;\right]}_{F}$) and backward ( ${\left[\;\right]}_{B}$) hemispheres. Here, integration over the angle θ needs to be performed in the intervals $F:0\leq \theta \leq \frac{\pi }{2}$ and $B:\frac{\pi }{2}\leq \theta \leq \pi$, respectively.

At this point, it is worth noticing that integration over the complete interval 0≤φ≤2π yields χ(α,β)=0. According to Eq. (5), only imaginary part of the expansion functions of the potential obey the asymmetry property (16) required for χ(α,β). We thus have to choose an integration interval for φ such that the first contribution in Eq. (19), which contains real parts of the expansion functions $V_{LM}^{\alpha ,\beta ,\gamma }(r)$, vanishes. Since it holds that^42^

$$V_{LM}^{\alpha ,\beta ,\gamma }(r)Y_{LM}(\theta ,\varphi )=V_{LM}^{\alpha ,\beta ,0}(r)Y_{LM}(\theta ,\phi =\varphi -\gamma ),$$ (20)

this can be achieved by choosing integration limits for the new angle ϕ=φ−γ as $-\frac{\pi }{2}\leq \phi \leq +\frac{\pi }{2}$ and simultaneously using expansion function $V_{LM}^{\alpha ,\beta ,0}(r)$ at γ=0 [as on the right-hand side of Eq. (20)]. As demonstrated in Appendix B, only expansion terms $V_{LM}^{\alpha ,\beta ,0}(r)$ with L+M=odd and with M=odd survive those integrations over θ and ϕ, respectively. According to Eq. (3), the characteristic χ(α,β), constructed over such expansion functions with L=even and M=odd, automatically obeys the required symmetry condition (15).

Finally, since outside the molecule the ionic potential has an asymptotic spherically symmetric Coulomb attraction ( $∼-\frac{1}{r}$), it makes sense to accumulate this property of the potential only within the molecule. Therefore, integration over the radial coordinate in Eq. (17) needs to be performed up to a “molecular radius” $R_{\text{mol}}$, at which all expansion functions saturate to their asymptotic behavior $V_{LM}^{\alpha ,\beta ,0}(r)∼\frac{1}{r^{L+1}}$. For molecules considered here, this radius $R_{\text{mol}}$ can be chosen in between 10 and 20 a.u. By introducing the accumulative quantities given by

$${\tilde{V}}_{LM}^{\alpha ,\beta }=\int_{0}^{R_{\text{mol}}}V_{LM}^{\alpha ,\beta ,0}(r)\;r^{2}\;dr,$$ (21)

we arrive at the following compact expression of the dichroic characteristic χ(α,β):

$$\begin{array}{c} \chi (\alpha ,\beta )=\frac{1}{\left|{\tilde{V}}_{00}\right|}\sum_{\overset{L_{\text{even}}}{M_{\text{odd}}\;>0}}^{}B_{LM}\;\text{Im}\left\{{\tilde{V}}_{LM}^{\alpha ,\beta }\right\}=\frac{1}{\left|{\tilde{V}}_{00}\right|}[-\sqrt{\frac{40}{3\pi }}\text{Im}\left\{{\tilde{V}}_{21}^{\alpha ,\beta }\right\}+\sqrt{\frac{1}{5\pi }}\text{Im}\left\{{\tilde{V}}_{41}^{\alpha ,\beta }\right\}\\ +\sqrt{\frac{7}{5\pi }}\text{Im}\left\{{\tilde{V}}_{43}^{\alpha ,\beta }\right\}\ldots ]. \end{array}$$ (22)

The explicit analytic expression for the numerical coefficients $B_{LM}$ is derived in Appendix B. Note also that the symmetric part of the potential, ${\tilde{V}}_{00}={\tilde{V}}_{00}^{\alpha ,\beta }$ (21), used in Eqs. (17) and (22) for the normalization, is independent of the molecular orientation.

As one can see from Eq. (22), the leading term $\text{Im}\left\{{\tilde{V}}_{21}^{\alpha ,\beta }\right\}$ of the dichroic asymmetry χ(α,β), which is proportional to $Y_{21}(\theta ,\varphi )∼\;\text{sin}\;\theta \;\text{cos}\;\theta \;e^{i\varphi }$, represents the simplest possible linear coupling of the rotational $\left[{\left(\text{sin}\;\theta e^{i\varphi }\right)}^{1}\right]$ and translational $\left[{\left(\text{cos}\;\theta \right)}^{1}\right]$ motions of the photoelectron in the forward-backward directions, provided by the ionic potential. If for a given molecular orientation the integral characteristic χ(α,β) is positive, then the potential couples on average a clockwise rotation more with the forward translation and counterclockwise more with the backward. One thus can expect that at this molecular orientation, more photoelectrons, ionized by the circularly polarized light of a positive helicity, will be emitted in the forward hemisphere. If at another orientation χ(α,β) is negative, one would expect more photoelectrons, ionized by the circularly polarized light of a positive helicity, to be emitted in the backward hemisphere. That is why these dichroic characteristics χ(α,β) and PECD(α,β) can directly be compared to each other.

## III. RESULTS AND DISCUSSION

We now analyze PECD(α,β) in the ionization of HOMO orbitals of several fixed-in-space chiral and achiral molecules and compare it to the dichroic characteristic χ(α,β) of the respective electronic potential of the HOMO^−1^ ion. Importantly, we use the same ionic potential for both, the PECD and χ(α,β) calculations.

### A. Chiral molecules

**FIG. 1.**
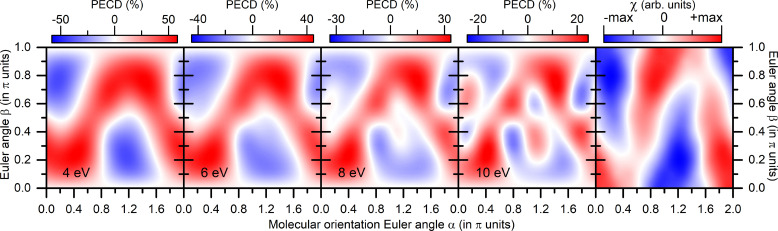
Theoretical results for the model methane-like chiral system from Ref. 15. Four panels from left to right: the dichroic asymmetry PECD(α,β) as function of the molecular orientation angles, computed for the photoelectron kinetic energies of ε=4, 6, 8, 10 eV (indicated in each panel). Rightmost panel: the dichroic characteristic of the potential χ(α,β). Note that the symmetric scales are used for the positive and negative values, and zero values are, thus, shown with white color.

We, first, consider a model methane-like chiral system introduced in our previous work.^15^ It is constructed over five point charges surrounded by localized spherically symmetric electron distributions. Details on the geometry and properties of the model can be found in Fig. 1 of Ref. 15. The calculations of the PECD were performed for four photoelectron kinetic energies of ε=4, 6, 8, 10 eV, as described in detail in this reference. Results of the present calculation of the dichroic asymmetry PECD(α,β) and of the respective dichroic characteristic of the ionic potential χ(α,β) are depicted in Fig. 1. To enable a direct comparison between those distributions, we employ symmetric-scale representations of all landscapes (i.e., set equal limits for positive and negative values), although maximal positive and negative absolute values differ from each other in every panel.

As one can see from Fig. 1, the PECD(α,β) distributions, computed at different photoelectron energies (indicated in the panels), have very similar landscapes as functions of the molecular orientation angles, albeit different (gradually decreasing with the energy) relative strength of the asymmetries. It is striking to see that the χ(α,β) landscape in the rightmost panel of this figure [which is mainly determined by the leading term $\text{Im}\left\{{\tilde{V}}_{21}^{\alpha ,\beta }\right\}$ in the expansion (22)] resembles the main trend and sign in the PECD(α,β) landscapes significantly. This means that, at those molecular orientations, where the χ(α,β) is maximal positive (negative), and the ionic potential couples a clockwise rotation with the forward (backward) translation in the most efficient way, the photoelectrons are indeed preferably emitted in the forward (backward) directions, as follows from the *ab initio* calculations of the PECD.

**FIG. 2.**
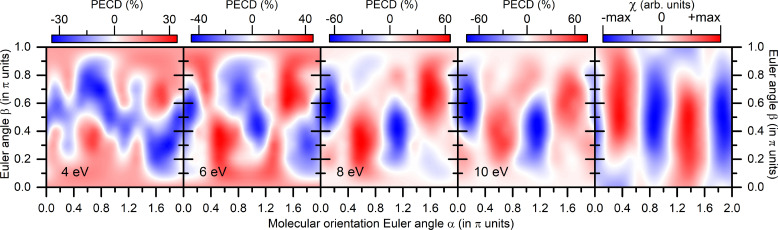
Theoretical results for R-fenchone molecules (see caption of Fig. 1 for details on data representation).

Further on, calculations of the PECD in R-fenchone molecules were performed as described in detail in our previous works.^37,38,41^ The dichroic asymmetry PECD(α,β), computed at the photoelectron energies of ε=4, 6, 8, 10 eV, and the dichroic characteristic of the ionic potential χ(α,β) are depicted in Fig. 2 in a similar way as in Fig. 1. As one can see, the computed PECD(α,β) landscapes evolve with the growth of the photoelectron energy (from the left to the right panels), establishing thereby a stable trend in the landscape. Such a sequence of negative–positive–negative–positive–negative vertical stripes (from left to right in each panel) is also present in the dichroic characteristic χ(α,β) in the rightmost panel of this figure at very similar orientation angles α. Here, again, this main trend is governed by the leading term $\text{Im}\left\{{\tilde{V}}_{21}^{\alpha ,\beta }\right\}$ in the expansion (22). Note that the PECD(α,β) landscape, computed for the smallest considered energy, differs from this main trend substantially (see discussion in Sec. IV for more details).

### B. Achiral molecules

In this subsection, we discuss two achiral molecules: carbon monoxide and ammonia. For the CO molecule, we used its equilibrium geometry from Ref. 47, restricted the single-center expansion with ℓ,|m|<17, and adjusted the local exchange potential Xα^48^ to equate the computed one-electron binding energy of the HOMO with its experimental value of 14.01 eV.^49^ According to the present definition of the enantiomers for achiral molecules (R=S), given at the very end of Sec. II A, the molecular $z^{\prime }$ axis coincides with the C=O bond. Because of this choice and also owing to the axial symmetry of the molecule, the molecular potential is independent of the orientation angles α and γ and also of the azimuthal angle $\varphi^{\prime }$. As a consequence, all expansion functions of the potential $V_{LM}^{\alpha ,\beta ,\gamma }(r)$ (2) are real-valued at each molecular orientation. Since the dichroic characteristic of the potential χ(α,β) is built in Eq. (22) of imaginary parts of the potential functions, it vanishes at all orientations, i.e., χ(α,β)=0. This means that a potential of axially symmetric molecules couples clockwise and counterclockwise rotations equally with the forward and backward translations. Indeed, the present calculations yield that the forward-backward asymmetries (10) are equivalent for the two circular polarizations of the ionizing light, $FB^{+}(\alpha ,\beta )=FB^{-}(\alpha ,\beta )$, and, as a result, the respective dichroic asymmetry vanishes as well: PECD(α,β)=0.

**FIG. 3.**
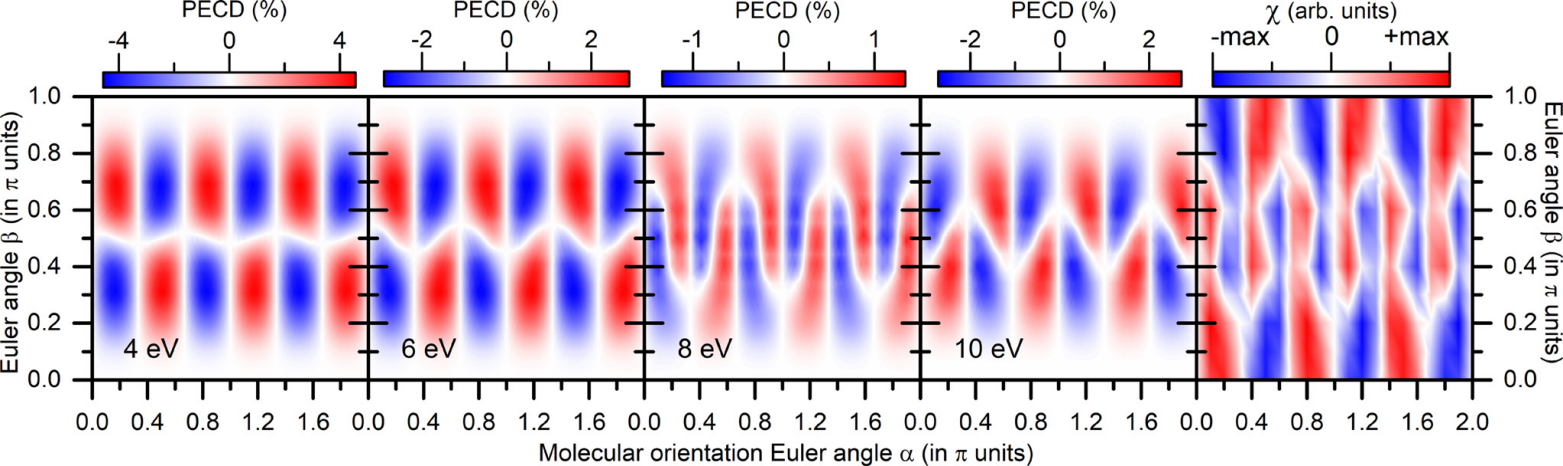
Theoretical results for NH_3_ molecules (see caption of Fig. 1 for details on data representation).

For the NH_3_ molecule, we used the equilibrium internuclear geometry from Ref. 50, employed the single-center expansion with ℓ,|m|<10, and adjusted the local exchange potential Xα to equate the computed one-electron binding energy of the HOMO with its experimental value of 10.85 eV.^49^ The molecular frame was set such that the nitrogen atom belongs to and points in a positive direction of the molecular $z^{\prime }$ axis, which is perpendicular to the plane built of three hydrogen atoms. In order to fulfill the condition R=S for this achiral molecule, all hydrogen atoms are located symmetrically with respect to the molecular $x^{\prime }z^{\prime }$-plane, and one of them with the coordinate $x^{\prime }>0$ has the coordinate $y^{\prime }=0$. Results of the present calculations are depicted in Fig. 3. As one can see from this figure, the computed dichroic asymmetry PECD(α,β) and characteristic χ(α,β) exhibit very similar landscapes, which are threefold periodic in the orientation angle α in the interval [0,2π], as also dictated by the molecular $C_{3v}$ symmetry. That is why the term $\text{Im}\left\{{\tilde{V}}_{21}^{\alpha ,\beta }\right\}$ in the expansion (22) vanishes by symmetry, and the landscape of χ(α,β) is governed by the leading terms $\text{Im}\left\{{\tilde{V}}_{41}^{\alpha ,\beta }\right\}$ and $\text{Im}\left\{{\tilde{V}}_{43}^{\alpha ,\beta }\right\}$. Contrary to the considered before chiral molecules, the maximal positive and negative absolute values in each landscape are equivalent (i.e., all landscapes are symmetric). The PECD(α,β) landscapes evolve with increase in the photoelectron energy, and that computed at 8 eV reproduces the χ(α,β) landscape to a great extent. However, the PECD(α,β) landscapes, computed for the photoelectron energies below 8 eV, exhibit opposite signs as compared to the χ(α,β) landscape, and the sign flips at the kinetic energy of 10 eV. Extended calculations showed that in between the photoelectron energies of 8 and 10 eV, there is a continuous transformation between these landscapes. This suggests that the coupling between the rotational and translational photoelectron motions should be energy dependent (see also below).

## IV. CONCLUSIONS AND OUTLOOK

The present theoretical work is a first step toward characterization of the electronic potential of the molecular ion and establishing its simplest possible enantiosensitive dichroic characteristic χ(α,β), which can be compared with the photoelectron circular dichroism of the fixed-in-space molecule PECD(α,β). This characteristic reveals that the physical mechanism responsible for the asymmetric coupling between the rotational and translational motions of a photoelectron, moving in the ionic potential, is governed by the imaginary parts of the ${\tilde{V}}_{LM}^{\alpha ,\beta }$ terms with L=even and M=odd values. The presently performed numerical applications to different chiral and achiral molecules suggest that this characteristic allows one to predict distinct molecular orientations, at which this mechanism works in the most efficient way. As a consequence, largest forward-backward asymmetries in the photoemission (i.e., largest PECD) can be expected at these orientations.

For achiral molecules, both the dichroic asymmetry PECD(α,β) and the characteristic of the ionic potential χ(α,β) exhibit landscapes, which are symmetric in their negative and positive parts and values, since a mirror image of an achiral molecule (which yields an opposite effect) can be created by a rotation. This means that achiral molecules couple on average any rotational motion equally with the forward and backward translations. As a consequence, averaging those landscapes over all molecular orientations (which models a situation of a freely rotating molecule in the gas phase) yields zero for both, PECD(α,β) and χ(α,β), as is also expected for the PECD. On the contrary, for chiral molecules, the PECD(α,β) landscapes are asymmetric in their negative and positive parts and values, and they average to different, usually non-zero, values of $\text{PECD}=2\beta_{1}$ for different photoelectron kinetic energies.

Although, for chiral molecules, the characteristic χ(α,β) exhibits asymmetric landscape as well, it always averages to zero, which can be shown analytically using properties of the Wigner rotation matrices.^42^ This is a direct consequence of the fact that χ(α,β) is built from a real-valued (although chiral) scalar quantity (the ionic potential). In addition, the characteristic χ(α,β) knows only about the sense of rotation, but nothing about the ionizing light itself and its photon energy. As a consequence, unlike the PECD(α,β), it is independent of the photoelectron kinetic energy. In spite of the shortcomings of the characteristic χ(α,β) discussed above, it can be understood in the following way. The energy-independent landscape χ(α,β), which averages to a zero value, can be recognized in the PECD(α,β) landscapes as a main template pattern. It represents an orientation-induced extrinsic contribution to the PECD and, thus, can be used for predictions of distinct molecular orientations, at which maximal PECD can be expected, without considering the photoionization process itself by *ab initio* theory. The interaction with the ionizing light, in turn, changes this pattern in different asymmetric ways for different photoelectron energies (more for lower kinetic energies). For molecules with intrinsic chirality, those changes in the PECD(α,β) do not average over molecular rotations to zero, yielding thereby non-zero PECD values for randomly oriented molecules.

We finally notice that in order to establish a dichroic enantiosensitive characteristic of the ionic potential, which on average over molecular orientations yields different non-zero values for different photoelectron energies, an energy-dependent chiral vector needs to be introduced to probe this chiral scalar. This, however, is outside the scope of the present work and is a task for future study.

## Data Availability

The data that support the findings of this study are available from the corresponding author upon reasonable request.

## APPENDIX A: EXPANSION COEFFICIENTS $b_{L}^{\pm }(\alpha ,\beta )$

In this appendix, an explicit dependence of the expansion coefficients $b_{L}^{\pm }(\alpha ,\beta )$ from Eq. (7) on the molecular orientation angles is derived using an analytical framework of our previous works.^51–55^

The photoelectron wave function in the continuum spectrum of energy $\varepsilon =k^{2}/2$ is defined by the following superposition of partial spherical waves with given angular momentum quantum numbers ℓ and $m^{\prime }$:^56^

$$\Psi_{{\vec{k}}^{\prime }}^{-}({\vec{r}}^{\;\prime })=\sum_{\ell m^{\prime }}{\left(i\right)}^{\ell }R_{\varepsilon \ell m^{\prime }}(r^{\prime })\;Y_{\ell m^{\prime }}(\theta^{\prime },\varphi^{\prime })\;Y_{\ell m^{\prime }}^{*}(\theta_{k}^{\prime },\varphi_{k}^{\prime }).$$ (A1)

Here, prime represents all quantities in the frame of a molecular reference, as computed at the initial orientation angles {α=0,β=0,γ=0}. For transparency, we incorporated the normalization coefficient and phases of the partial waves in their radial parts. In the molecular frame of reference, the photoionization matrix elements for an emission of the partial wave $\varepsilon \ell m^{\prime }$ through the absorption of one photon of polarization $k^{\prime }$ by an initial electronic orbital $\Phi_{0}$ reads

$$d_{\varepsilon \ell m^{\prime }k^{\prime }}=\langle R_{\varepsilon \ell m^{\prime }}Y_{\ell m^{\prime }}|d_{k^{\prime }}|\Phi_{0}\rangle ,$$ (A2)

where $d_{k^{\prime }}$ is the electric-dipole transition operator, and k=0 stays for the linear and k=±1 for two circular polarizations.

Proceeding along the lines of Ref. 57, we first transform the electric-dipole operator $d_{q}$ from the laboratory to molecular frame with the help of the Wigner rotation matrices $D_{ij}^{\ell }$,

$$d_{q}=\sum_{k^{\prime }}D_{k^{\prime }q}^{1}(\alpha ,\beta ,\gamma )\;d_{k^{\prime }},$$ (A3)

and the partial photoelectron waves from Eq. (A1) in the opposite way,

$$Y_{\ell m^{\prime }}(\theta_{k}^{\prime },\varphi_{k}^{\prime })=\sum_{m}D_{m^{\prime }m}^{\ell *}(\alpha ,\beta ,\gamma )\;Y_{\ell m}(\theta_{k},\varphi_{k}).$$ (A4)

Using Eqs. (A1)–(A4), the total transition amplitude for the ionization of a molecule, which is fixed in space at arbitrary orientation angles {α,β,γ}, by the absorption of one photon with polarization q=±1 can be written in the following form:

$$\begin{array}{c} T_{\varepsilon }^{q}(\alpha ,\beta ,\gamma ,\theta_{k},\varphi_{k})=\sum_{\ell m^{\prime }k^{\prime }m}{\left(-i\right)}^{\ell }D_{k^{\prime }q}^{1}(\alpha ,\beta ,\gamma )\\ \times D_{m^{\prime }m}^{\ell *}(\alpha ,\beta ,\gamma )\;Y_{\ell m}(\theta_{k},\varphi_{k})\;d_{\varepsilon \ell m^{\prime }k^{\prime }}. \end{array}$$ (A5)

Taking the modulus square of the amplitude (A5), reducing the product of two spherical functions, and averaging over the orientation angle γ (over the emission angle $\varphi_{k}$, see also respective discussion in Sec. II B), we arrive at the expansion (7) for the two-dimensional photoemission probability. The explicit analytic expression for the expansion coefficients $b_{L}^{q}(\alpha ,\beta )$ with q=±1 reads

$$\begin{array}{c} b_{L}^{q}(\alpha ,\beta )=\sum_{\ell_{1}m_{1}^{\prime }k_{1}^{\prime }}\sum_{\ell_{2}m_{2}^{\prime }k_{2}^{\prime }}\sum_{m}{\left(i\right)}^{\ell_{1}+\ell_{2}}\;{\left(-1\right)}^{\ell_{1}+m}\\ \times \sqrt{2\ell_{1}+1)(2\ell_{2}+1)}\frac{2L+1}{4\pi }\\ \times \left(\begin{array}{ccc} \ell_{1} & \ell_{2} & L\\ 0 & 0 & 0 \end{array}\right)\left(\begin{array}{ccc} \ell_{1} & \ell_{2} & L\\ m & -m & 0 \end{array}\right)\\ \times D_{k_{1}^{\prime }q}^{1}\;D_{k_{2}^{\prime }q}^{1*}\;D_{m_{1}^{\prime }m}^{\ell_{1}*}\;D_{m_{2}^{\prime }m}^{\ell_{2}}d_{\varepsilon \ell_{1}m_{1}^{\prime }k_{1}^{\prime }}\;d_{\varepsilon \ell_{2}m_{2}^{\prime }k_{2}^{\prime }}^{*}, \end{array}$$ (A6)

where dependence of the rotation matrices on orientation angles is not shown for brevity.

## APPENDIX B: EXPANSION COEFFICIENTS $B_{LM}$

In this appendix, an explicit expression for the expansion coefficients $B_{LM}$ from Eq. (22) is derived. For this purpose, we substitute the second term on the right-hand side of Eq. (19) in the nominators of Eq. (17), perform integration over the radial coordinate by Eq. (21), and set the integration limits for the angles θ and ϕ, as discussed in the main text after Eqs. (19) and (20), respectively. Taking the real part from the spherical functions represented via the associated Legendre polynomials,^42^ $Y_{LM}(\theta ,\phi )=\sqrt{\frac{2L+1}{4\pi }\frac{(L-M)!}{(L+M)!}}\;P_{LM}(\text{cos}\;\theta )e^{iM\phi }$, we obtain

$$\begin{array}{c} \chi (\alpha ,\beta )=\frac{2}{\left|{\tilde{V}}_{00}\right|}\sum_{L,M>0}\text{Im}\left\{{\tilde{V}}_{LM}^{\alpha ,\beta }\right\}\\ \times \sqrt{\frac{2L+1}{4\pi }\frac{(L-M)!}{(L+M)!}}\left[\int_{-\frac{\pi }{2}}^{+\frac{\pi }{2}}\;\text{cos}(M\phi )d\phi \right]\\ \times \left[\int_{0}^{\frac{\pi }{2}}P_{LM}(\text{cos}\;\theta )\;\text{sin}\;\theta \;d\theta -\int_{\frac{\pi }{2}}^{\pi }P_{LM}(\text{cos}\;\theta )\;\text{sin}\;\theta \;d\theta \right]. \end{array}$$ (B1)

The first integration in Eq. (B1) over the angle ϕ yields

$$\int_{-\frac{\pi }{2}}^{+\frac{\pi }{2}}\;\text{cos}(M\phi )d\phi =\{\begin{array}{l} ll{\left(-1\right)}^{\frac{M-1}{2}}\frac{2}{M}\;,\;\;\;M=\text{odd},\\ 0\;,\;\;\;M=\text{even}. \end{array}$$ (B2)

This restricts the expansion (B1) to the positive odd values of *M*. The second integration in Eq. (B1) over the angle θ yields

$$\begin{array}{c} \int_{0}^{\frac{\pi }{2}}P_{LM}(\text{cos}\;\theta )\;\text{sin}\;\theta \;d\theta -\int_{\frac{\pi }{2}}^{\pi }P_{LM}(\text{cos}\;\theta )\;\text{sin}\;\theta \;d\theta \\ =\{\begin{array}{cc} 2\int_{0}^{1}P_{LM}(\zeta )\;d\zeta , & L+M=\text{odd},\\ 0, & L+M=\text{even}, \end{array} \end{array}$$ (B3)

with the following analytical solution for the integral:^58^

$$\begin{array}{c} \int_{0}^{1}P_{LM}(\zeta )\;d\zeta =\frac{{\left(-1\right)}^{M}\;\pi \;(L+M)!\;{\left(2\right)}^{-2M-1}}{\Gamma \left(\frac{M+1}{2}\right)\Gamma \left(\frac{M+3}{2}\right)(L-M)!}\\ \times {\;}_{3}F_{2}(\frac{L+M+1}{2},\frac{M-L}{2},\frac{M+2}{2};M+1,\frac{M+3}{2};1). \end{array}$$ (B4)

In Eq. (B4), Γ stands for the gamma function of a real argument and ${}_{3}F_{2}$ is the generalized hypergeometric function.^59^ As one can see, the integration (B3) restricts the expansion (B1) to the terms with L+M=odd. As a consequence of both integrations, only coefficients ${\tilde{V}}_{LM}^{\alpha ,\beta }$ with L=even and M=odd remain in the expansion (B1). Substituting all those analytical results in Eq. (B1), we arrive at the compact expression (22) of the characteristic χ(α,β) with the expansion coefficients $B_{LM}$ given explicitly by

$$\begin{array}{c} B_{LM}={\left(-1\right)}^{\frac{M+1}{2}}\frac{{\left(2\right)}^{-2M+1}}{M}\sqrt{\pi \;(2L+1)\frac{(L+M)!}{(L-M)!}}\\ \times \frac{{\;}_{3}F_{2}(\frac{L+M+1}{2},\frac{M-L}{2},\frac{M+2}{2};M+1,\frac{M+3}{2};1)}{\Gamma \left(\frac{M+1}{2}\right)\Gamma \left(\frac{M+3}{2}\right)}. \end{array}$$ (B5)
